# MRI Markers and Functional Performance in Patients With CIS and MS: A Cross-Sectional Study

**DOI:** 10.3389/fneur.2018.00718

**Published:** 2018-08-29

**Authors:** Ludwig Rasche, Michael Scheel, Karen Otte, Patrik Althoff, Annemieke B. van Vuuren, Rene M. Gieß, Joseph Kuchling, Judith Bellmann-Strobl, Klemens Ruprecht, Friedemann Paul, Alexander U. Brandt, Tanja Schmitz-Hübsch

**Affiliations:** ^1^NeuroCure Clinical Research Center, Charité – Universitätsmedizin Berlin, Corporate Member of Freie Universität Berlin, Humboldt-Universität zu Berlin, and Berlin Institute of Health, NeuroCure Cluster of Excellence, Berlin, Germany; ^2^Department of Neuroradiology, Charité – Universitätsmedizin Berlin, Corporate Member of Freie Universität Berlin, Humboldt-Universität zu Berlin, and Berlin Institute of Health, Berlin, Germany; ^3^Motognosis GmbH, Berlin, Germany; ^4^VU University Medical Center, Amsterdam, Netherlands; ^5^Department of Neurology, Charité – Universitätsmedizin Berlin, Corporate Member of Freie Universität Berlin, Humboldt-Universität zu Berlin, and Berlin Institute of Health, Berlin, Germany; ^6^Experimental and Clinical Research Center, Charité - Universitätsmedizin Berlin Corporate Member of Freie Universität Berlin, Humboldt-Universität zu Berlin, and Berlin Institute of Health and Max Delbrück Center for Molecular Medicine, Berlin, Germany; ^7^Department of Neurology, University of California, Irvine, Irvine, CA, United States

**Keywords:** multiple sclerosis, clinically isolated syndrome, atrophy, thalamus, clinical outcomes, MSFC, functional performance

## Abstract

**Introduction:** Brain atrophy is a widely accepted marker of disease severity with association to clinical disability in multiple sclerosis (MS). It is unclear to which extent this association reflects common age effects on both atrophy and function.

**Objective:** To explore how functional performance in gait, upper extremities and cognition is associated with brain atrophy in patients with Clinically Isolated Syndrome (CIS) and relapsing-remitting MS (RRMS), controlling for effects of age and sex.

**Methods:** In 27 patients with CIS, 59 with RRMS (EDSS ≤3) and 63 healthy controls (HC), 3T MRI were analyzed for T2 lesion count (T2C), volume (T2V) and brain volumes [normalized brain volume (NBV), gray matter volume (NGMV), white matter volume (NWMV), thalamic volume (NThalV)]. Functional performance was measured with short maximum walking speed (SMSW speed), 9-hole peg test (9HPT) and symbol digit modalities test (SDMT). Linear regression models were created for functional variables with stepwise inclusion of age, sex and MR imaging markers.

**Results:** CIS differed from HC only in T2C and T2V. RRMS differed from HC in NBV, NGMV and NThalV, T2C and T2V, but not in NWMV. A strong association with age was seen in HC, CIS and RRMS groups for NBV (*r* = −0.5 to −0.6) and NGMV (*r* = −0.6 to −0.8). Associations with age were seen in HC and RRMS but not CIS for NThalV (*r* = −0.3; *r* = −0.5), T2C (*r*_*s*_ = 0.3; *r*_*s*_ = 0.2) and T2V (*r*_*s*_ = 0.3; *r*_*s*_ = 0.3). No effect of age was seen on NWMV. Correlations of functional performance with age in RRMS were seen for SMSW speed, 9HPTand SDMT (*r* = −0.27 to −0.46). Regression analyses yielded significant models only in the RRMS group for 9HPT, SMSW speed and EDSS. These included NBV, NGMV, NThalV, NWMV, logT2V, age and sex as predictors. NThalV was the only MRI variable predicting a functional measure (9HPT_r_) with a higher standardized beta than age and sex (*R*^2^ = 0.36, *p* < 1e-04).

**Conclusion:** Thalamic atrophy was a stronger predictor of hand function (9HPT) in RRMS, than age and sex. This underlines the clinical relevance of thalamic atrophy and the relevance of hand function as a clinical marker even in mildly disabled patients.

## Introduction

Multiple sclerosis (MS) is a chronic inflammatory and neurodegenerative disease of the central nervous system and the primary neurological disease causing disability in young adults (1). The number and location of hyperintense lesions on T2 weighted MRI (lesion load) have classically been used as a surrogate of inflammatory disease activity and an outcome in clinical studies of disease-modifying drugs. Other imaging features like global and focal brain and spinal cord atrophy have been described to occur even early in the course of MS (2, 3) and are now widely accepted as outcome parameters of disease progression (4, 5). In contrast to markers of inflammatory disease activity, such volume changes are thought to reflect the neurodegenerative trait of the disease (6, 7). Special focus has been given to thalamic atrophy, since it occurs early and progresses consistently during the disease course and thus might serve as an applicable MRI-marker in neuroprotective trials (3, 8). Clinically, MS may present with a variety of neurological signs and symptoms with impaired locomotor function among the most frequently reported and disabling symptoms (9–11). The Expanded Disability Status Scale (EDSS) is considered the gold standard and most widely used clinical scale to describe MS related disability (12, 13). Its organization on a non-linear scale makes structure-function correlation analyses difficult. Further, as a compound measure it does not allow for an analysis of specific symptoms of MS with respect to imaging findings. Quantitative functional testing has among other approaches been proposed as a more appropriate and sensitive measure of disability progression in MS (14, 15). The Multiple Sclerosis Functional Composite (MSFC), which has been developed for this purpose, assesses clinical dimensions of arm, leg and cognitive function in terms of performance and processing speed (16, 17). Whilst the original MSFC included the Paced Auditory Serial Addition Test (PASAT) as a measure of cognitive processing speed, this has largely been replaced by the Symbol Digits Modalities Test (SDMT) (18). A recent review series of the Multiple Sclerosis Outcome Assessments Consortium reported on the components of this modified MSFC (SDMT, nine-hole peg test and timed 25-foot walk) and proposed them as the relevant measures in these functional domains (19–21). Another recent development aims to make instrumented motor testing clinically more feasible. In this respect, different groups have described visual perceptive computing (VPC), which makes use of the Microsoft Kinect® infrared depth sensor, as a promising assessment tool in different neurological disorders (22–28). The VPC derived short maximum speed walk has been proven as highly comparable to timed 25-foot walk results in a previous study in 83 people with RRMS and 57 healthy subjects. Both measures were highly correlated (*r*_*p*_ = 0.75, *p* < 0.001 in healthy subjects and *r*_*p*_ = 0.78, *p* < 0.001 in RRMS) and mean between-method difference was 0.001 ± 0.2 m/s (28).

The association of MRI atrophy markers and disability in MS is well-established (29, 30). Whilst the existing body of literature generally confirms such association in MS cohorts with significant disability, evidence on functional correlates of brain atrophy in CIS and early RRMS patients is less compelling (31, 32). The scope of this study was therefore to explore the relation of imaging markers of disease severity with the above-mentioned hallmark parameters of functional decline in a cohort of mildly disabled CIS and RRMS patients (EDSS ≤3).

We expected to reproduce the occurrence of CNS tissue volume loss even in people with CIS or RRMS and mild disability, i.e., in absence of pronounced functional impairment. We further assumed age effects to be seen on both, global or compartmental volume loss and quantitative functional testing. The main hypothesis was, that an effect of structural MRI could be shown on functional performance, independent of effects of age and sex. There was no a priori hypothesis concerning the relation of motor function with lesion burden. An analysis of this parameter was included to allow inferences on the impact of inflammatory activity as opposed to markers of neurodegeneration.

## Methods

### Study population

We included clinical and MRI Data from different observational studies in clinically isolated syndrome (CIS) and MS at the Neurocure Clinical Research Center, Charité–Universitätsmedizin Berlin (EA1/182/10, EA1/077/11, and EA1/163/12). The studies were approved by the local ethics commitee of Charité–Universitätsmedizin Berlin in accordance with the Declaration of Helsinki in its current applicable state. All participants provided written informed consent for participation and publication of results. For restriction to mildly affected patients, we chose a cut-off of EDSS score of ≤3 (33), but no restriction was made regarding disease duration. Data of 27 people with CIS and 59 people with relapsing-remitting type of MS (RRMS), diagnosed according to the revised McDonald criteria from 2010 (34) and 63 healthy subjects (HC), matched as a group with respect to age distribution and sex, were included in this *post-hoc* analysis. No restrictions were made as to type of disease modifying treatment. Data were collected between September 2011 and December 2017 according to standardized protocols. All study visits were planned in stable remission of relapse, defined as at least 3 months after onset of most recent relapse. On average there was a delay between motor and imaging assessments of 0.6 days (SD ± 1.9) in CIS and 2.9 (SD ± 9.5) in RRMS patients. Demographic and clinical data are shown in Table 1.

**Table 1:** Demographics, clinical and MRI data reported as mean along with standard deviation or median (range).

	**HC (*n* = 63)**	**CIS (*n* = 27)**	**RRMS (*n* = 59)**	**Between group comparison HC and RRMS**	
Age (y)	35.3 ± 9.9	34.3 ± 7.0	37.5 ± 9.7	*t* = −1.21	*p* = 0.23
Gender (F/M)	40/23	19/8	33/26	*X*^2^ = 0.72	*p* = 0.39
Time since onset (y)		2.3 ± 1.4	4.1 ± 2.7		
NBV (ml)	1,586.2 ± 72.7	1,599.8 ± 72.0	1,553.6 ± 67.6	***t*** = **2.57**	***p*** = **0.01**
NGMV (ml)	846.2 ± 57.4	858.3 ± 56.3	823.7 ± 55.0	***t*** = **2.21**	***p*** = **0.03**
NThalV (ml)	21.5 ± 1.6	21.7 ± 1.1	20.6 ± 1.8	***t*** = **3.01**	***p*** = **0.003**
NWMV (ml)	740.0 ± 37.3	741.5 ± 38.2	729.9 ± 33.2	*t* = 1.59	*p* = 0.11
T2C (n)	3 (0–98)	9 (0–92)	27(1–199)	***W*** = **349.5**	***p*** = **9.57e-15**
T2V (ml)	0.3 ± 0.8	1.2 ± 2.6	4.3 ± 5.3	***W*** = **215**	***p*** < **2.2e-16**
EDSS median (range)		1.5 (0–3.0)	1.5 (0–3.0)		
EDSS Score		*n*	*n*		
0		7	10		
1		5	13		
1.5		7	13		
2.0		7	10		
2.5		0	7		
3.0		1	6		
SMSW speed (m/s)		1.8 ± 0.2	1.7 ± 0.3		
9HPT_dom_ (s)		17.8 ± 2.3	18.7 ± 3.1		
9HPT_nondom_ (s)		19.1 ± 2.6	20.5 ± 6.9		
9HPT_r_ (1/mean perf. time)		0.055 ± 0.006	0.053 ± 0.008		
SDMT (*n*)		53 ± 6.8	56.6 ± 11.7		

*Test statistics from unpaired t-test, Chi-squared or Wilcoxon test as indicated. Values in bold denote significance*.

### Clinical assessment and VPC motor measures

Experienced trained raters assessed EDSS, 9HPT and SDMT. Short maximum walking speed was quantified by a VPC system consisting of Microsoft Kinect® Version 2 video and infrared camera (Kinect SDK, Microsoft Corp., Redmont WA, USA) along with custom-written software (PASS-MS, Motognosis Labs Version 1.2–1.4, Motognosis GmbH, Berlin) as described previously (22). Short maximum speed walk yields the SMSW speed in m/s as the mean of three trials of <5 meters length, known to equal closely the T25FW performance speed (28).

### MRI acquisition and imaging measures

All study subjects were scanned using a 3 Tesla Siemens TimTrio scanner according to standardized protocols. Native 3D T1-weighted magnetization prepared rapid gradient-echo (MPRAGE) images (resolution 1 × 1 × 1 mm^3^; TR = 1,900 ms, TE = 3.03 ms, TI = 900 ms, flip angle 9°) were put into standard space and lesion in-filled with lesion segmentation masks for structural measurements. Native 3D T2-weighted fluid attenuated inversion recovery (FLAIR) images (resolution 1 × 1 × 1 mm^3^; TR = 6,000 ms, TE = 388 ms, TI = 2,100 ms, flip angle 120°) were used for T2-weighted lesion segmentation using the lesion prediction algorithm as implemented in the LST toolbox version 2.0.15 (35) and then co-registered to standard space. MPRAGE and lesion masks were edited using ITK-SNAP (36) manually.

Whole brain T2 lesion burden is given as T2 total lesion volume (T2V) and the number of T2 lesions (T2C), including cerebellum and brainstem. T2C and T2V were not normalized, since an influence of brain size on lesion count / volume deemed unlikely.

Volumes for whole brain, gray and white matter, were estimated using the lesion in-filled MPRAGE images by fsl SIENAX (37). Thalamic volume was calculated using fsl FIRST (38), the sum of both sides was used for further calculations. All volumes were normalized using the normalization factor provided from SIENAX, which accounts for variable brain sizes.

### Statistical analysis

All statistical analyses were performed using R (Version 3.3.2) via R-Studio (Version 1.0.136). Imaging and clinical parameters were tested for normal distribution per group of CIS, RRMS and HC, using the one-sample Kolmogorov Smirnoff test and calculation of skewness. A *p*-value >0.05 in the Kolmogorov Smirnoff test and an absolute amount of skewness of <1 was set as criterion for normal distribution. For skewed parameters (T2C and T2V), common log transformed values were used for regression analyses. In order to include patients and controls without cerebral lesions (CIS *n* = 1, RRMS *n* = 0, HC *n* = 18) in correlation analysis, the logT2C was given a value at the lower border within the observed distribution (0). Similarly, the logT2V of patients and controls without lesions were given a logT2V value at the lower border of the observed distribution (−2). Comparison between CIS, RRMS and HC was performed using the Wilcoxon Test or Students *t*-test as indicated in Table 1. Three group comparisons were carried out using Anova or Kruskall-Wallis test accordingly. Individual performance on quantitative functional testing was classified using previously published own normative data for SMSW speed (28). More precisely, expected normal SMSW speed was calculated for each individual according to the following formula:

$$\text{SMSWspeed }\left(\frac{\text{m}}{\text{s}}\right)\text{= −0}.\text{008 }\times \text{age }\left(\text{years}\right)\text{ + 2}.\text{161}$$

This formula was derived from a univariate regression analysis for the age effect on gait speed in a cohort of *n* = 57 HC subjects with an average SMSW speed of 1.83 m/s ± 0.26 (28). From there, individual observed SMSW speeds in CIS and RRMS were classified as pathologic if they were beyond a threshold of 1.95-fold standard deviation below the expected normal value.

For 9HPT, the performance time of each hand was defined as pathologic if values were beyond a threshold of 1.95-fold standard deviation around age- and sex-related normative means (39, 40). For further correlation and regression analysis, we used the transformed 9HPT score (9HPT_r_) as suggested in the MSFC manual (41) which combines both hands' performance. SDMT performance was classified as pathologic if values were below a threshold of 1.95-fold standard deviation with respect to published age- and sex-related norms (42).

Effects of age on MRI findings and parameters of functional performance were explored by Pearson correlation except for T2C and T2V, where Spearman correlation was used. A possible confounding effect of disease duration (time since onset) was explored by correlation of age vs. time since onset. This approach showed a significant association that was based on two relatively old subjects (50 and 55 years of age) with a time since onset of 13 and 15 years (Supplementary Figure 1). However, both subjects were only mildly affected in the assessed clinical domains and thus are not expected to determine the age effects in our regression models. When excluding these subjects only a trend remained between age and time since onset. We therefore decided to treat age as an independent variable in our analysis.

To answer the main objective, functional performance parameters were put in separate linear regression models and fitted with a set of predictors in a hierarchical succession, using the lm.beta function for linear regression objects and the stargazer package in R-Studio (43, 44). We performed a stepwise inclusion of age, sex and the respective MRI parameter. A similar approach was also applied with EDSS as dependent variable. This part of the analysis was done only on CIS and RRMS data. *P*-values <0.05 were defined as significant for exploratory parts of analysis. For regression models, we applied Bonferroni correction per MRI parameter (*n* = 6), which set significance level to *p* < 0.008.

## Results

Demographic, clinical and MRI data are presented in Table 1. CIS and RRMS groups differed in numbers and were imbalanced with respect to sex with a F/M ratio in CIS of 2.4 and in RRMS of 1.3 and disease duration with time since onset of 2.3 and 4.1 years, respectively. Regarding the EDSS, there was no difference in the median score, but a higher proportion of patients with EDSS >2 in RRMS (*n* = 13, 22%) in comparison to one patient (4%) in the CIS group.

### Brain volumetric measures

**Figure 1:**
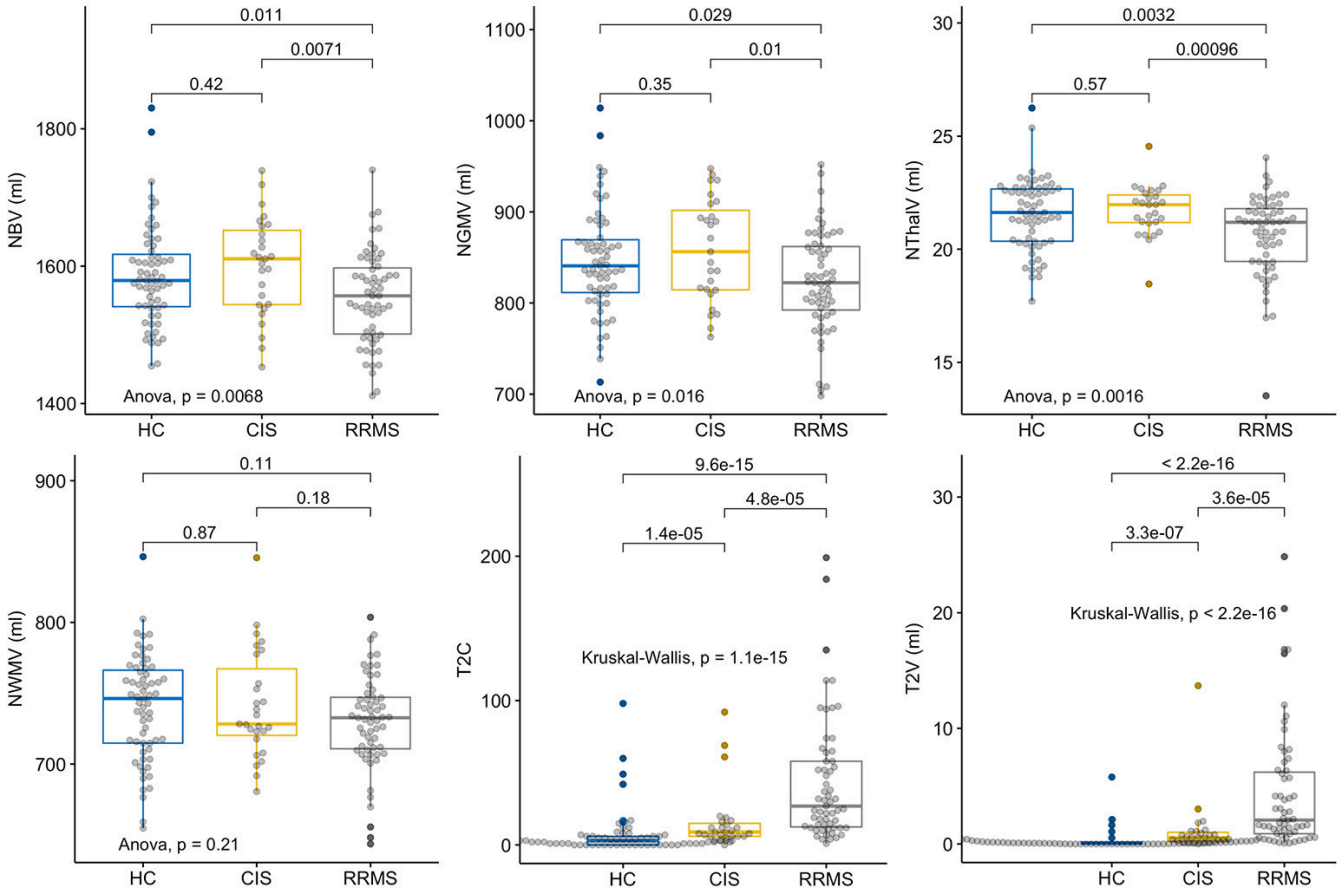
Group comparisons of MRI parameters. Displayed are the respective MRI Parameters in healthy controls (HC), patients with clinically isolated syndrome (CIS) and relapsing-remitting multiple sclerosis (RRMS). The horizontal line represents the median, the box represents the upper and lower quartiles, whiskers represent the 1.5-fold IQR, outliers are encircled.

CIS subjects showed no significant differences compared to HC regarding the brain volumetric measures (Figure 1). There was a significant group difference with lower values for RRMS compared to HC and CIS for NBV (*t* = 2.57, *p* = 0.01; *t* = 2.82, *p* = 0.007), NGMV (*t* = 2.21, *p* = 0.03; *t* = 2.67, *p* = 0.01), NThalV (*t* = 3.01, *p* = 0.003; *t* = 3.43, *p* = 0.0009; Figure 1). No significant between-group difference was observed for NWMV (*p* = 0.1–0.2), although visual inspection of the distributions suggested a tendency toward higher volumes in HC in respect to CIS and RRMS (Figure 1). As expected, CIS and RRMS differed from HC in T2C and T2V (Figure 1). CIS patients had significantly increased T2C (*W* = 359.5, *p* < 0.0001) and T2V (*W* = 273, *p* < 0.0001) compared to HC, while T2C and T2V were higher in RRMS compared to HC (*W* = 349.5, *p* < 0.0001; *W* = 215, *p* < 0.0001) and CIS (*W* = 359.5, *p* = 0.0005; *W* = 352, *p* = 0.0004; Figure 1). There was a significant number of T2 lesions in single HC subjects that were reevaluated by a radiologist (M.S.) in order to exclude relevant comorbidity. Lesions were all classified as unspecific white matter lesions and no controls had to be excluded.

### Clinical parameters

For the SMSW Speed, none of the 27 CIS patients and only three of 59 RRMS patients (5%) had an abnormal maximum walking speed (defined as <1.95 times normative SD below the individually estimated age-related norm). Regarding 9HPT, 3 of 27 (11%) CIS patients showed performance times >1.95 times normative SD above the age- and sex-related norms in the dominant or non-dominant hand. The combined proportion of patients showing an abnormal value in either one or both hands accounted to 5 of 27 (19%). In RRMS, 10 of 56 (18%) showed a pathologic performance time in the dominant hand. A pathologic performance time in the non-dominant hand was seen in 9 of 56 (16%) and the variance was higher than in the dominant hand. The combined proportion of RRMS patients showing abnormal value in either one or both hands accounted to 14 of 56 (25%). For three individuals 9HPT data was not available. Regarding the SDMT, one of 27 (4%) CIS and four of 59 (7%) RRMS patients showed abnormally low numbers of responses with respect to age- and sex-adjusted norms.

### Effects of age

**Figure 2:**
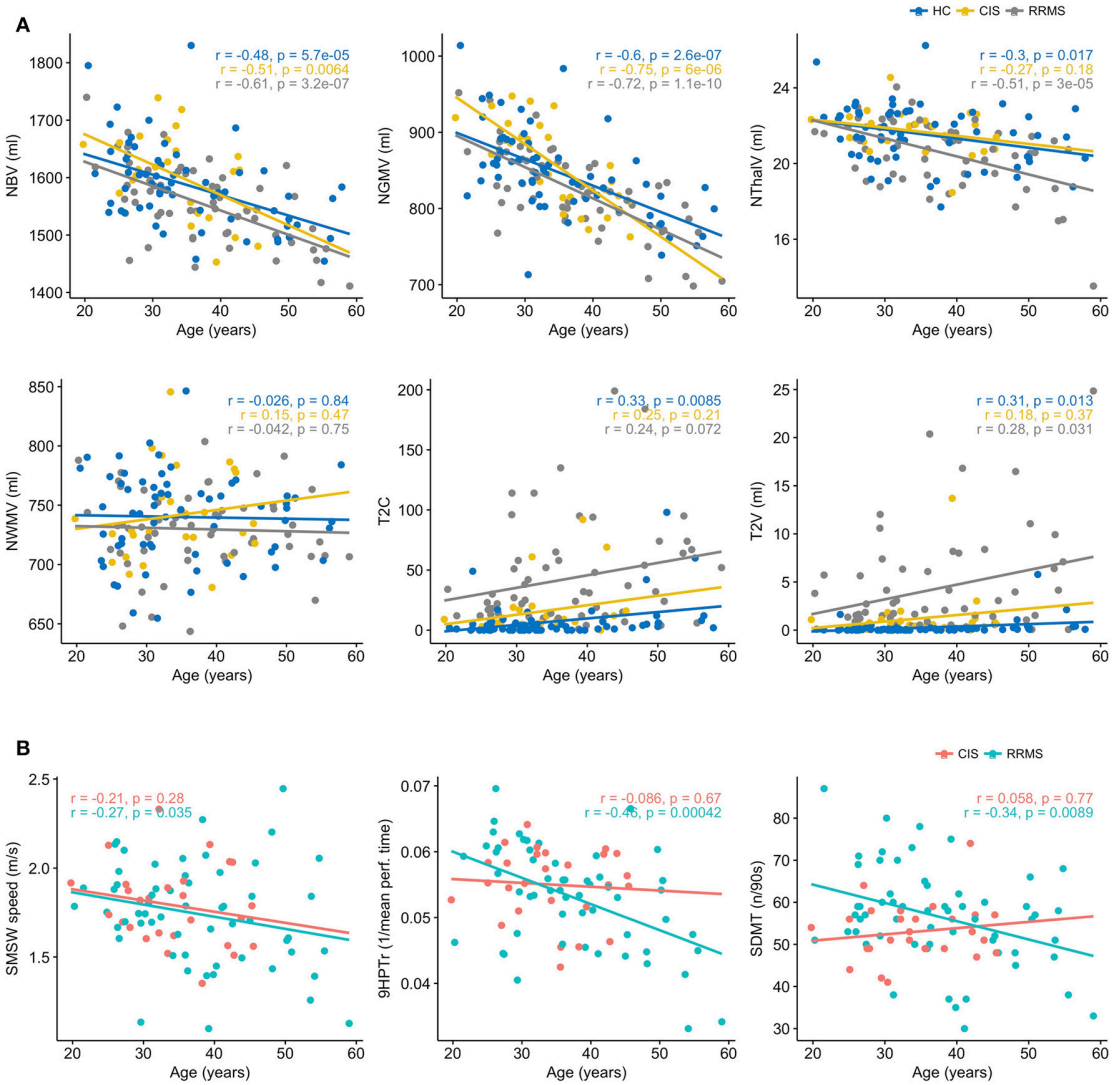
**(A)** Age dependency of MRI parameters in healthy controls (HC), patients with clinically isolated syndrome (CIS) and relapsing-remitting multiple sclerosis (RRMS). Displayed are the Pearson's *r* and *p*, except for T2C and T2V, where Spearmans rho and *p* are displayed. **(B)** Age dependency of functional performance in patients with clinically isolated syndrome (CIS) and relapsing-remitting multiple sclerosis (RRMS). Displayed are the Pearsons *r* and *p*.

In bivariate correlations, we saw an association with age in HC, CIS and RRMS for NBV (*r* = −0.48, *p* < 0.0001; *r* = −0.51, *p* = 0.006; *r* = −0.61, *p* < 0.0001) and NGMV (*r* = −0.60, *p* < 0.0001; *r* = −0.75, *p* < 0.0001; *r* = −0.72, *p* = < 0.0001). In HC and even more so in RRMS, NThalV (*r* = −0.3, *p* = 0.017; *r* = −0.51, *p* < 0.0001) also showed an association with age. While a similar slope regarding NThalV and Age in HC was also observed in CIS patients, this remained non-significant (*r* = −0.27, *p* = 0.18). No correlation with age was seen for NWMV in either group. Regarding T2C, only HC showed a significant association with age (*r*_*s*_ = 0.33, *p* = 0.008). No association was seen in CIS (*r*_*s*_ = 0.25, *p* = 0.21), while a trend was seen in RRMS patients (*r*_*s*_ = 0.24, *p* = 0.072). Likewise, T2V was associated with age in HC (*r*_*s*_ = 0.31, *p* = 0.013 and RRMS (*r*_*s*_ = 0.28, *p* = 0.031), but not in CIS (*r*_*s*_ = 0.18, *p* = 0.37; Figure 2). Regarding logT2C and logT2V an association with age was seen only in HC (*r* = 0.4, *p* = 0.001; *r* = 0.4, *p* = 0.0005), while no such association was observed in CIS (*r* = 0.26, *p* = 0.19; *r* = 0.14, *p* = 0.49) or RRMS (*r* = 0.16, *p* = 0.23; *r* = 0.07, *p* = 0.6), respectively (not shown). Concerning clinical parameters in CIS, no significant correlations with age were found for SMSW speed (*r* = −0.21, *p* = 0.28), 9HPT_r_ (*r* = −0.09, *p* = 0.67) or SDMT (*r* = 0.06, *p* = 0.77)_._ Concerning clinical parameters in RRMS, significant correlations with age were found for SMSW speed (*r* = −0.27, *p* = 0.035), 9HPT_r_ (−0.46, *p* = 0.0004) and SDMT (*r* = −0.34, *p* = 0.009).

### Regression analyses

In the CIS subgroup, no significant multivariate models were found (Supplementary Table 1). In the RRMS subgroup however, several significant multivariate linear regression models were found that explained little (<20%) to moderate (20–35%) proportions of the variance in the dependent clinical variable:

With SMSW speed as the dependent variable, age alone accounted for a marginal proportion in the variance (5.5%). This proportion increased to 19.1% after adding sex into the model, probably due to larger decline with age in females (Supplementary Figure 2). Among MRI parameters, the NWMV had the largest relative effect on SMSW speed with a standardized beta (std. beta) of 0.275 (Table 2). This was however outweighed by sex (std. beta 0.284), while age had a std. beta of −0.225. The overall model had a *R*^2^ of 0.26 and retained significance after correction for multiple testing. Weaker relative effects on SMSW speed were also seen for NBV (std. beta 0.238) and NThalV (std. beta 0.248), although both parameters' effects were outweighed by sex (std. beta 0.373 and 0.408).

**Table 2:** Regression models in patients with relapsing-remitting multiple sclerosis (RRMS) for functional performance measures as dependent variable and stepwise inclusion of age, sex, and the volumetric MRI markers.

	**Dependent Variables**											
	**SDMT**			**9HPTr**			**SMSW speed**			**EDSS**		
Age	−0.361	−0.361	−0.209	−0.491***	−0.488***	−0.365***	−0.235***	−0.237***	−0.093***	0.419***	0.419***	0.319***
	*p* = 0.016	*p* = 0.016	*p* = 0.262	*p* < 0.0001	*p* < 0.0001	*p* < 0.0001	*p* < 0.0001	*p* < 0.0001	*p* < 0.0001	*p* < 0.0001	*p* < 0.0001	*p* < 0.0001
sexM		0.044	0.049		−0.164***	−0.165***		0.368***	0.373***		0.081	0.078
		*p* = 0.989	*p* = 0.987		*p* < 0.0001	*p* < 0.0001		*p* < 0.0001	*p* < 0.0001		*p* = 0.714	*p* = 0.726
NBV			0.251***			0.206***			0.238***			−0.164***
			*p* < 0.0001			*p* < 0.0001			*p* < 0.0001			*p* < 0.0001
*R*^2^	0.130	0.132	0.172	0.241	0.268	0.295	0.055	0.191	0.226	0.176	0.182	0.199
*F*	8.550*	4.270	3.804	17.128**	9.682**	7.253**	3.341	6.598*	5.361*	12.163**	6.249*	4.566*
Age	−0.361	−0.361	−0.149	−0.491***	−0.488***	−0.407***	−0.235***	−0.237***	−0.181***	0.419***	0.419***	0.366***
	*p* = 0.016	*p* = 0.016	*p* = 0.498	*p* < 0.0001	*p* < 0.0001	*p* < 0.0001	*p* < 0.0001	*p* < 0.0001	*p* < 0.0001	*p* < 0.0001	*p* < 0.0001	*p* < 0.0001
sexM		0.044	0.106		−0.164***	−0.140***		0.368***	0.384***		0.081	0.066
		*p* = 0.989	*p* = 0.972		*p* < 0.0001	*p* < 0.0001		*p* < 0.0001	*p* < 0.0001		*p* = 0.714	*p* = 0.780
NGMV			0.295***			0.113***			0.078***			−0.074***
			*p* < 0.0001			*p* < 0.0001			*p* < 0.0001			*p* < 0.0001
*R*^2^	0.130	0.132	0.170	0.241	0.268	0.273	0.055	0.191	0.193	0.176	0.182	0.185
*F*	8.550*	4.270	3.754	17.128**	9.682**	6.513**	3.341	6.598*	4.394*	12.163**	6.249*	4.157
Age	−0.361	−0.361	−0.255	−0.491***	−0.488***	−0.300***	−0.235***	−0.237***	−0.110***	0.419***	0.419***	0.310***
	*p* = 0.016	*p* = 0.016	*p* = 0.143	*p* < 0.0001	*p* < 0.0001	*p* < 0.0001	*p* < 0.0001	*p* < 0.0001	*p* < 0.0001	*p* < 0.0001	*p* < 0.0001	*p* < 0.0001
sexM		0.044	0.077		−0.164***	−0.107***		0.368***	0.408***		0.081	0.047
		*p* = 0.989	*p* = 0.980		*p* < 0.0001	*p* < 0.0001		*p* < 0.0001	*p* < 0.0001		*p* = 0.714	*p* = 0.834
NThalV			0.207			0.356***			0.248***			−0.212*
			*p* = 0.828			*p* < 0.0001			*p* < 0.0001			*p* = 0.004
*R*^2^	0.130	0.132	0.163	0.241	0.268	0.356	0.055	0.191	0.234	0.176	0.182	0.214
*F*	8.550*	4.270	3.562	17.128**	9.682**	9.563***	3.341	6.598*	5.605*	12.163**	6.249*	5.003*
Age	−0.361	−0.361	−0.356	−0.491***	−0.488***	−0.487***	−0.235***	−0.237***	−0.225***	0.419***	0.419***	0.411***
	*p* = 0.016	*p* = 0.016	*p* = 0.018	*p* < 0.0001	*p* < 0.0001	*p* < 0.0001	*p* < 0.0001	*p* < 0.0001	*p* < 0.0001	*p* < 0.0001	*p* < 0.0001	*p* < 0.0001
sexM		0.044	0.006		−0.164***	−0.241***		0.368***	0.284***		0.081	0.135
		*p* = 0.989	*p* = 0.999		*p* < 0.0001	*p* < 0.0001		*p* < 0.0001	*p* < 0.0001		*p* = 0.714	*p* = 0.560
NWMV			0.121			0.217***			0.275***			−0.174***
			*p* = 0.009			*p* < 0.0001			*p* < 0.0001			*p* < 0.0001
*R*^2^	0.130	0.132	0.146	0.241	0.268	0.309	0.055	0.191	0.259	0.176	0.182	0.210
*F*	8.550*	4.270	3.124	17.128**	9.682**	7.739**	3.341	6.598*	6.409**	12.163**	6.249*	4.868*

Given are the standardized beta coefficients, R^2^ and respective F-Statistic, * denotes significance after correction for multiple testing.

* *p < 0.008*,

** *p < 0.001*,

*** *p < 0.0001*.

Several models with 9HPT_r_ as the dependent variable remained significant when correcting for multiple testing. Age alone explained 24.1% of the variance in 9HPT_r_. When adding sex, this increased to 26.8%. Among the MRI parameters, NBV and NThalV explained relevant proportions in the variance of 9HPT_r_ when age and sex effects were taken into account (Table 2). For NBV, the std. beta (0.206) was smaller than the amount of the std. beta for age (−0.365) and larger than that for sex (−0.165) with a model *R*^2^ of 0.30 (*p* < 0.0001). NThalV was the sole imaging parameter in this analysis that had a higher std. beta (0.365) than the amount of the std. beta for age (−0.300) and sex (−0.107), the *R*^2^ was highest among all models with 0.36 (*p* < 0.0001).

For SDMT as the dependent variable, only a trend was seen regarding age. Sex played no significant part in explaining SDMT variance. Of the imaging parameters, NBV and NGMV had the largest std. beta (0.251 and 0.295, both *p* < 0.0001) but both overall models had a small *R*^2^ and were non-significant.

LogT2V explained part of the variance of SMSW speed with a beta of −0.191 but the effects of age (std. beta −0.224) and sex (std. beta 0.368) were stronger (model *R*^2^ 0.227). There were no sex differences in respect to age-related change in logT2V, therefore the high standardized beta for sex in the model stems mainly from its influence on age-related decline in SMSW speed (Supplementary Figure 2). LogT2C and T2V explained a marginal proportion of the variance in 9HPT_r_ (beta −0.054; −0.150), but only the model including T2V remained significant after correction for multiple testing (Supplementary Table 1). No effect on SDMT was seen for both lesional parameters.

Regarding the EDSS as dependent variable, several models remained significant after correction for multiple testing. Age alone was the strongest predictor with an *R*^2^ of 0.178. After inclusion of Sex, the *R*^2^ increased only slightly to 0.182. Among the MRI parameters, NBV, NGMV, NWMV and NThalV showed relative effects in predicting EDSS variance. The amount of the std. beta for NBV and NWMV was larger than for sex (NBV std. beta = −0.164, sex std. beta = 0.078; NWMV std. beta = −0.174, sex std. beta = 0.135). NThalV had the largest relative effect with a std. beta of −0.212, while the std. beta for sex was 0.047 and the model *R*^2^ amounted to 0.214. However, none of the MRI parameters had a larger std. beta than age, which remained the strongest predictor for EDSS (std. beta 0.319–0.411).

## Discussion

In the current study, we explored the relation of structural MRI parameters (T2 lesions, compartmental brain atrophy) with different domains of functional performance in mildly affected patients with CIS and RRMS (EDSS ≤3). Our aim was to further clarify the role of brain atrophy in predicting functional outcomes and to differentiate between physiologic effects of age and disease specific effects. Although we consider the same pathophysiologic mechanisms to be active in CIS and RRMS we could not confirm differences to HC in our (smaller) CIS group. When comparing compartmental MRI volumetric data of CIS and RRMS with HC, differences on group level where found for NBV, NGMV and NThalV between RRMS vs. HC and RRMS vs. CIS, but not in CIS compared to HC. No overall group differences were observed for NWMV in total. T2 lesion load and T2 lesion volume showed a significant difference on group level as expected in CIS and RRMS compared to HC. Regarding CIS, the lack of group differences in respect to HC for all brain volume measures studied, might be explained by the inclusion criterion of only mild clinical disability for this analysis. In a longitudinal study by Fisniku et al. (45) patients with an EDSS >3 had a significantly higher gray matter atrophy than patients with an EDSS ≤3. This selection criterion and a diagnosis of CIS also resulted in a rather short disease duration with a mean time since onset in this group of 2.3 years. A limiting factor for the group comparison of the CIS and HC subgroups as well as linear regression analysis in the CIS group was the relatively small number (*n* = 27) of CIS. However, our observations suggest, that compartmental atrophy in the CIS subgroup was not pronounced and would probably need more sensitive imaging methods. Accordingly, we observed only small numbers of pathologic functional performance in the CIS group and structural-functional regression analysis could not show marked associations with either of the analyzed predictors for CIS (Supplementary Table 2).

Although effects of age and time since disease onset cannot be completely disentangled, we concluded from exploratory analysis (see Methods section) that it is rather unlikely that the effects of age determined in our study are mere reflections of disease duration. Further, the notion of similar age effects on compartmental volumes in both, CIS, RRMS and HC, strongly argues for a predominant effect of age. In the RRMS group, several compartmental volumes with significant group differences were observed in our cohort, which confirms that neurodegeneration has taken place at an early stage of disease. Group differences were most pronounced in NThalV (percent of difference 4.3%), which confirms that early neurodegeneration is most visible in this structure. This is in line with previous data suggesting that thalamic atrophy occurs early and at consistent rates throughout the disease course (3, 8, 46). Our results indicate further that thalamic atrophy is a promising marker especially in mildly disabled patients. Accordingly, the imaging marker with largest group difference—NThalV-displayed stronger age effects in the RRMS group than in CIS and HC.

Considering maximum walking speed in the RRMS group, the prevalence of pathologic values was very low (4%). This might again be explained by the selection of our cohort based on EDSS that accordingly only includes subjects that are at least considered fully ambulatory based on maximum walking distance (47). As expected, bivariate correlations suggested a weak decline of SMSW speed with age. Several significant multivariate regression models with SMSW speed as the dependent variable including age, sex and the various MRI parameters were found. Of note, NWMV had the largest relative impact on SMSW speed among all imaging markers, whereas the strongest predictor was sex at the disadvantage of women. This might be attributed to a larger body size and step length in males but remains a surprising finding. Different studies have reported on associations of atrophy markers with gait speed [see Rocca et al. for review (29)]. For example, decreases in maximum walking speed (T25FW) were seen with progression of NWMV atrophy as well as atrophy in subcortical gray matter volumes, including thalamus, but not NGMV, which would be in line with our findings, if sex was not included in the regression model (48). However, in partial correlations adjusting for age and sex in their study, only caudate and putamen volume retained a significant effect in predicting T25FW speed. In another study, Shiee et al. reported on a negative correlation of T25FW performance time with normalized thalamic volume [*r* = −0.32 (*p* = 0.01)], and normalized brainstem volume [*r* = −0.31 (*p* = 0.01)] in partial correlations adjusting for age and sex, while they found no significant associations with lesion load, normalized gray matter or white matter volume in a cohort of 60 MS patients (mean age 43), with mixed subtypes (median EDSS 2.7 (0–6.5) (49). Van de Pavert et al. on the other hand have found no associations with regional deep gray matter volume and maximum walking speed or 9HPT in a voxel-based analysis on a total of 80 MS patients (median EDSS 5.75 (0–8.5). However, they reported on associations of maximum walking speed with cerebellar volume and atrophy in the post-central gyrus, as well as with gray matter lesion volume in these regions (50). In sum, although conflicting results exist on correlations of maximum speed gait performance with deep gray matter volumes (that may in part be due to use of performance time vs. performance speed), there is no report of a correlation for maximum gait speed with global gray matter volume even in large cohorts of more disabled patients which is in line with our findings. The tendency for an association of NWMV with maximum gait speed has also been reported by Motl et al. (48). If white matter atrophy is a disease specific characteristic in MS has been a matter of debate. A difficulty for the analysis of volume related functional association poses its natural pattern of volume change, since white matter volume shows a volume increase until the age of approximately 40 in healthy adults and subsequently declines (51). This non-linearity hampers longitudinal structural-functional analysis. Evidence is also growing, that functional and microstructural methodologies to assess white matter integrity might be more suitable than volumetric analyses to describe subtle structural-functional associations in this compartment (52). However, Shiee et al. have reported that patients with lower white matter volume tend to have more disability (49) and Howard et al. showed a significant difference in NWMV in those patients who required ambulatory assistance vs. those who do not while the difference was even greater for NGMV and NBV (53). This implies that largest effects of structural-functional analyses with NWMV might be expected in later disease stages (or older subjects). However, maximum walking speed is obviously no applicable parameter of functional performance in later disease stages, as most subjects will be unable to perform. In our RRMS cohort with mild disability, in contrast, analysis with maximum walking speed might be limited by rather narrow range of observed performance. Other studies in early MS have captured abnormalities of gait despite normal maximum walking speed (54). This and the suggestion of non-linearity also for this parameter (55) cast some doubt on the use of maximum walking speed as a marker of functional decline even in fully ambulatory patients, despite its otherwise excellent metric properties (19) and current recommendations.

Impairment of hand function was the most prevalent deficit among the three hallmark functional domains within our RRMS cohort. Based on published reference values, 24% of our patients showed pathologic 9HPT performance times in one or both hands without obvious preponderance of either hand. This proportion is surprisingly close to published proportions of self-reported mild to severe hand dysfunction (26%) at a disease duration of 3 years in a large sample of MS patients (56) but proportions can be expected in up to 66% in unstratified MS cohorts (57). It has to be noted that a less conservative cut-off of 1 SD from age and sex related norms is often used (58). When applied to our population, the proportion of patients with a pathologic 9HPT score rises to 41%. The high proportion of pathologic 9HPT performance supports the clinical relevance of upper limb function when evaluating mildly affected MS patients for functional impairment which contrasts common perception, that hand testing becomes more relevant in those where gait tests are no longer applicable. Across the MS spectrum, the most consistent association of MRI and 9HPT performance has been reported for cerebellar atrophy (50, 59, 60), which was not looked at in this study. Besides the association with cerebellar atrophy, D'Ambrosio et al. also found a weak association with NBV and cerebral T2LV but not with NGMV or NWMV in univariate linear regression (59). Shiee et al. have reported a correlation of 9HPT performance with thalamus volume in a cohort of 60 MS patients with mixed subtypes (median EDSS 2.7) (49), although they also found significant associations with other MRI structures. With averaged 9HPT performance time (from both hands) as the dependent variable, they reported negative partial correlations for Thalamic volume [*r* = −0.35 (*p* = 0.005)], White matter volume [*r* = −0.45 (*p* < 0.001)] and cerebral volume fraction [*r* = −0.46 (*p* < 0.001)], as well as a positive correlation with lesion load [*r* = 0.34 (*p* = 0.008)], when accounting for age and sex. Similar to maximum gait speed performance, the comparison of studies using 9HPT are hampered by the fact that performance is reported in different ways (21). We have used the transformed 9HPT_r_ score as described in the MSFC manual, which does not take into account the sidedness of the actual performance. It is however unlikely, that this approach influences the correlation analyses *per se*, since neurodegeneration is not known to take place with particular asymmetry.

Cognitive processing speed, as assessed with SDMT, was in the pathological range in only 7% of RRMS patients. This again underlines the relatively mild affection of our cohort and poses some limitation to regression analyses, as discussed with SMSW speed. According to Kister et al., the self-perceived cognitive impairment at 3 years of disease is higher with up to 38% of patients reporting mild to moderate cognition problems (56). However, as has been pointed out by the authors, mismatch between patient reported and objective outcomes in different functional domains may be explained by a diverging patient awareness of deficits and this holds particularly true for the cognitive domain (61, 62). Concerning the structural-functional association, no significant models where found for SDMT. This contrasts findings in MS cohorts with a wider spectrum of disability in which a relation of SDMT to whole brain measures (brain volume, T2 lesion volume) was evident (63), while evidence of such relationship in patients with early relapsing-remitting MS or CIS is sparse (63, 64). Consistent with our findings, more recent studies in early RRMS or CIS cohorts (32, 65, 66) were unable to confirm correlations of SDMT performance with volumetric brain measures while Bisecco et al. have reported on thalamic atrophy as an independent and additional contributor to SDMT variance in a cross-sectional analysis controlled for sex and age of 125 RRMS patients (mean age 37 years, median EDSS 2) (67). However, they used a different analytic approach (voxel-based morphometry analysis) and the MS group differed more significantly in several compartmental volumes (NBV, NGMV and NWMV) from the HC group, suggesting higher overall atrophy compared to our CIS and RRMS groups. In sum, while associations of SDMT with atrophy of gray matter and thalamus, as well as T2LC and T2LV are evident in MS patients with a wider spectrum of disability, this relation is less frequently reported in CIS or early RRMS patients, which is in line with our findings.

As the key finding of this study we report that variance in 9HPT performance speed in mildly disabled patients with RRMS was significantly predicted by thalamic volume, more so than by age or sex. The functional domain of hand dexterity as well as thalamus volume obviously show early involvement within the disease course of MS, as differences to HC were most prominent among all functional and imaging parameters evaluated in this study and results beyond normal range were also observed in 19% of CIS patients. The structural-functional relationship between thalamic atrophy and 9HPT performance can be put into a pathophysiological context when taking into account that the thalamus is highly involved in motor control, integrating information from the cortex, the basal ganglia and the cerebellum (68). It seems possible that the correlation of 9HPT with thalamic atrophy in our study mirrors the correlations of 9HPT performance with cerebellar atrophy reported in other studies, but this awaits further investigation. Regarding the other MRI parameters their effect on the performance measures in the cognitive and gait domain was outweighed by age and to some extent by sex. This age dependency of MRI and functional parameters has thus to be thoroughly accounted for, especially when evaluating cross sectional data in early and or mildly affected MS patients.

Our study is limited due to its cross-sectional character and due to the low overall variance in the parameters of functional impairment. Further, as functional performance was only tested in CIS and RRMS, differences to physiological performance as well as age dependency of these parameters can only be inferred from published normative datasets. While thalamic atrophy has been credited functional importance for gait function and cognitive performance in MS patients previously, our data support its role also for manual dexterity. Our results underline the relevance of both, thalamic atrophy and quantitative functional testing of upper limb function in CIS and RRMS patients with mild disability.

## Data availability statement

The raw data supporting the conclusions of this manuscript will be made available by the authors, without undue reservation, to any qualified researcher.

## Author contributions

LR, TS-H, AB, KR, MS, and FP contributed conception and design of the study; MS, LR, PA, KO, JK, AvV, JB-S, and RG were involved in acquisition and organization of clinical and/or MRI data; LR performed the statistical computations and wrote the first draft of the manuscript; TS-H, AB, and MS verified the analytical methods and supervised the finalization of the manuscript; All authors contributed to manuscript revision, read and approved the submitted version.

### Conflict of interest statement

JK received conference registration fees from Biogen and financial research support from Krankheitsbezogenes Kompetenznetzwerk Multiple Sklerose (KKNMS), unrelated to this work. JB-S received speaking fees and travel grants from Bayer Healthcare, sanofi-aventis/Genzyme, Biogen and Teva Pharmaceuticals, unrelated to the present scientific work. KR was supported by the German Ministry of Education and Research (BMBF/KKNMS, Competence Network Multiple Sclerosis) and has received research support from Novartis and Merck Serono as well as speaking fees and travel grants from Guthy Jackson Charitable Foundation, Bayer Healthcare, Biogen Idec, Merck Serono, sanofi-aventis/Genzyme, Teva Pharmaceuticals, Roche and Novartis, unrelated to this work. FP reports research grants and speaker honoraria from Bayer, Teva, Genzyme, Merck, Novartis, MedImmune and is member of the steering committee of the OCTIMS study (Novartis), all unrelated to this work. AB is founder and holds shares of Motognosis GmbH and nocturne-oct. He is named as inventor on several patent applications describing serum biomarkers for MS, perceptive visual computing for tracking of motor dysfunction and OCT image analysis. KO is a shareholder of Motognosis GmbH and named as inventor on patent applications describing perceptive visual computing for tracking of motor dysfunction. TS-H received research grants from Ipsen Pharma and speaker honoraria from Rölke pharma, all unrelated to this work. LR, RG, MS, PA, and AvV have nothing to disclose.

## Abbreviations

CIS: clinically isolated syndrome
EDSS: expanded disability status scale
HC: healthy controls
logT2C: logarithmic T2 lesion count
logT2V: logarithmic T2 lesion volume
MRI: magnetic resonance imaging
MS: multiple sclerosis
MSFC: multiple sclerosis functional composite
NBV: normalized brain volume
NGMV: normalized gray matter volume
NThalV: normalized thalamic volume
NWMV: normalized white matter volume
RRMS: relapsing-remitting MS
SDMT: symbol digit modalities test
SMSW: short maximum speed walk
T2C: T2 lesion count
T2V: T2 lesion volume
9HPT: nine-hole peg test
9HPT_r_: transformed 9HPT score.
