# Supplementary material for: MRI Markers and Functional Performance in Patients With CIS and MS: A Cross-Sectional Study
Source: Front Neurol. 2018 Aug 29;9:718. doi: 10.3389/fneur.2018.00718 (PMC6123531; doi:10.3389/fneur.2018.00718)
Supplement: Supplementary file 1 [file Data_Sheet_1.docx]

Supplementary Material

MRI Markers and Functional Performance in Patients with CIS and MS: A Cross-Sectional Study

Ludwig Rasche, Michael Scheel, Karen Otte, Patrik Althoff, Annemieke Van Vuuren, Rene M. Gieß, Joseph Kuchling, Judith Bellmann-Strobl, Klemens Ruprecht, Friedemann Paul, Alexander U. Brandt, Tanja Schmitz-Hübsch^*^

*** Correspondence:**Corresponding Author
[tanja.schmitz-huebsch@charite.de](mailto:tanja.schmitz-huebsch@charite.de)

# Supplementary Figures and Tables

##
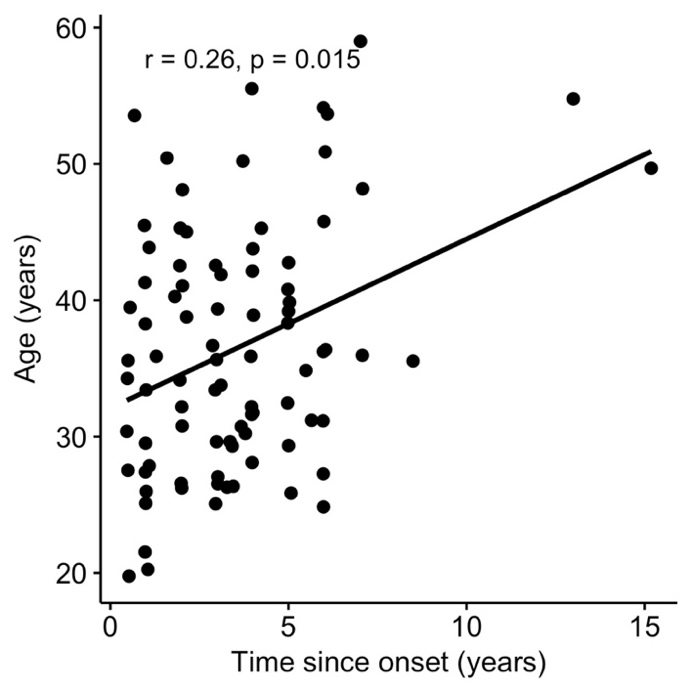
Supplementary Figures

Supplementary Figure 1. Association of time since onset and age in all patients. Given are the spearman´s rho and p. When excluding the two individuals with a time since onset >10years, only a trend remained for this correlation (r_s_=0.21, p=0.053).


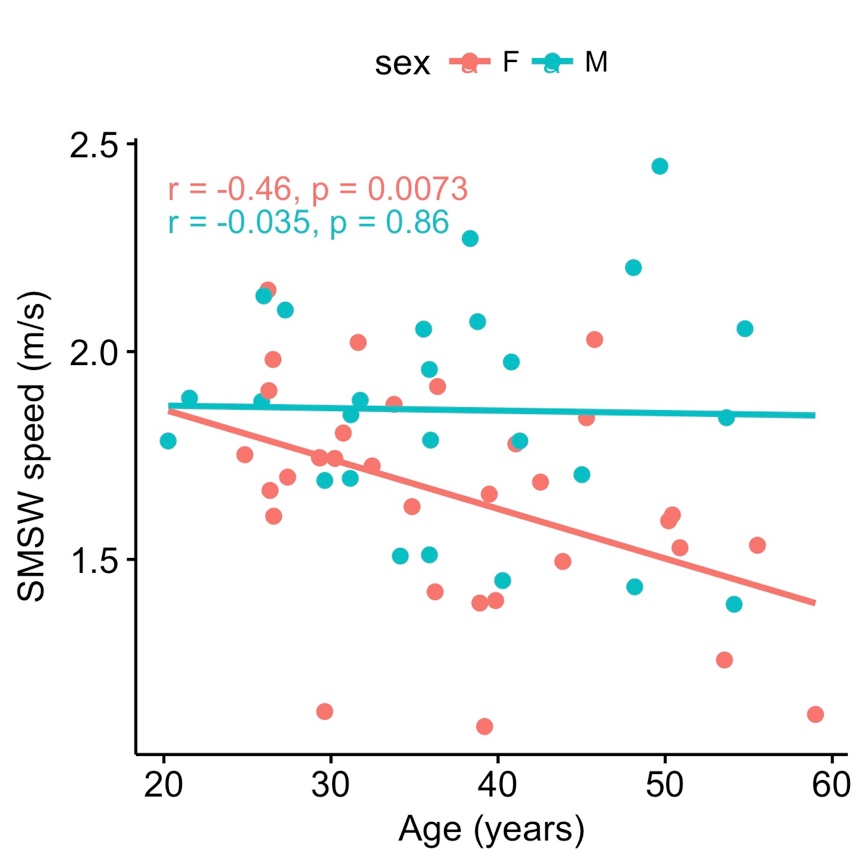


**Supplementary Figure 2.** Association of short maximum walking speed (SMSW) and age in patients with relapsing-remitting multiple sclerosis (RRMS), stratified by sex. Given are the Pearsons r and p.

## Supplementary Tables

|  | *Dependent variables:* | | | | | | | | | | | |
| --- | --- | --- | --- | --- | --- | --- | --- | --- | --- | --- | --- | --- |
|  | SDMT | | | 9HPTr | | | SMSW speed | | | EDSS | | |
| Age | *-0.361* | *-0.361* | *-0.360* | *-0.491^***^* | *-0.488^***^* | *-0.481^***^* | *-0.235^***^* | *-0.237^***^* | *-0.214^***^* | *0.419^***^* | *0.419^***^* | *0.385^***^* |
|  | p = 0.016 | p = 0.016 | p = 0.019 | p < 0.0001 | p < 0.0001 | p < 0.0001 | p < 0.0001 | p < 0.0001 | p < 0.0001 | p < 0.0001 | p < 0.0001 | p < 0.0001 |
| SexM |  | *0.044* | *0.044* |  | *-0.164^***^* | *-0.159^***^* |  | *0.368^***^* | *0.377^***^* |  | *0.081* | *0.069* |
|  |  | p = 0.989 | p = 0.989 |  | p < 0.0001 | p < 0.0001 |  | p < 0.0001 | p < 0.0001 |  | p = 0.714 | p = 0.753 |
| logT2C |  |  | *-0.010* |  |  | *-0.054^***^* |  |  | *-0.147* |  |  | *0.213* |
|  |  |  | p = 0.998 |  |  | p < 0.0001 |  |  | p = 0.048 |  |  | p = 0.374 |
| R^2^ | 0.130 | 0.132 | 0.132 | 0.241 | 0.268 | 0.270 | 0.055 | 0.191 | 0.212 | 0.176 | 0.182 | 0.227 |
| F | 8.550^*^ | 4.270 | 2.798 | 17.128^**^ | 9.682^**^ | 6.426^**^ | 3.341 | 6.598^*^ | 4.926^*^ | 12.163^**^ | 6.249^*^ | 5.372^*^ |
|  | SDMT | | | 9HPTr | | | SMSW speed | | | EDSS | | |
| Age | *-0.361* | *-0.361* | *-0.365* | *-0.491^***^* | *-0.488^***^* | *-0.482^***^* | *-0.235^***^* | *-0.237^***^* | *-0.224^***^* | *0.419^***^* | *0.419^***^* | *0.405^***^* |
|  | p = 0.016 | p = 0.016 | p = 0.016 | p < 0.0001 | p < 0.0001 | p < 0.0001 | p < 0.0001 | p < 0.0001 | p < 0.0001 | p < 0.0001 | p < 0.0001 | p < 0.0001 |
| SexM |  | *0.044* | *0.039* |  | *-0.164^***^* | *-0.147^***^* |  | *0.368^***^* | *0.385^***^* |  | *0.081* | *0.064* |
|  |  | p = 0.989 | p = 0.990 |  | p < 0.0001 | p < 0.0001 |  | p < 0.0001 | p < 0.0001 |  | p = 0.714 | p = 0.771 |
| logT2V |  |  | *0.050* |  |  | *-0.150^***^* |  |  | *-0.191^**^* |  |  | *0.197* |
|  |  |  | p = 0.984 |  |  | p < 0.0001 |  |  | p = 0.001 |  |  | p = 0.278 |
| R^2^ | 0.130 | 0.132 | 0.135 | 0.241 | 0.268 | 0.290 | 0.055 | 0.191 | 0.227 | 0.176 | 0.182 | 0.221 |
| F | 8.550^*^ | 4.270 | 2.856 | 17.128^**^ | 9.682^**^ | 7.075^**^ | 3.341 | 6.598^*^ | 5.376^*^ | 12.163^**^ | 6.249^*^ | 5.195^*^ |
|  | ^*^p^**^p^***^p<1e-04 | | | | | | | | | | | |

**Supplementary Table 1.** Regression models in RRMS for functional performance measures as dependent variable and stepwise inclusion of Age, Sex and logarithmic T2 count and volume. Given are the *standardized beta coefficients*, R^2^ and respective F-Statistic, ^*^ is signifying significance after correction for multiple testing.

|  | *Dependent variables:* | | | | | | | | | | | |
| --- | --- | --- | --- | --- | --- | --- | --- | --- | --- | --- | --- | --- |
|  | SDMT | | | 9HPTr | | | SMSW speed | | | EDSS | | |
| Age | *0.151* | *0.144* | *0.241* | *-0.073^***^* | *0.007^***^* | *0.282^***^* | *-0.197^***^* | *-0.086^***^* | *-0.252^***^* | *0.125^***^* | *0.089^**^* | *0.024* |
|  | p = 0.435 | p = 0.497 | p = 0.342 | p < 0.0001 | p < 0.0001 | p < 0.0001 | p < 0.0001 | p < 0.0001 | p < 0.0001 | p < 0.0001 | p = 0.001 | p = 0.441 |
| SexM |  | *0.022* | *-0.009* |  | *-0.228^***^* | *-0.318^***^* |  | *-0.319^*^* | *-0.265^*^* |  | *0.103* | *0.124* |
|  |  | p = 0.995 | p = 0.998 |  | p < 0.0001 | p < 0.0001 |  | p = 0.002 | p = 0.007 |  | p = 0.794 | p = 0.761 |
| NBV |  |  | *0.169^***^* |  |  | *0.477^***^* |  |  | *-0.289^***^* |  |  | *-0.113^***^* |
|  |  |  | p < 0.0001 |  |  | p < 0.0001 |  |  | p < 0.0001 |  |  | p < 0.0001 |
| R^2^ | 0.023 | 0.023 | 0.044 | 0.005 | 0.051 | 0.212 | 0.039 | 0.128 | 0.187 | 0.016 | 0.025 | 0.034 |
| F | 0.586 | 0.287 | 0.349 | 0.133 | 0.644 | 2.059 | 1.011 | 1.764 | 1.764 | 0.398 | 0.307 | 0.269 |
|  | SDMT | | | 9HPTr | | | SMSW speed | | | EDSS | | |
| Age | *0.151* | *0.144* | *0.231* | *-0.073^***^* | *0.007^***^* | *0.352^***^* | *-0.197^***^* | *-0.086^***^* | *-0.442^***^* | *0.125^***^* | *0.089^**^* | *0.033* |
|  | p = 0.435 | p = 0.497 | p = 0.453 | p < 0.0001 | p < 0.0001 | p < 0.0001 | p < 0.0001 | p < 0.0001 | p < 0.0001 | p < 0.0001 | p = 0.001 | p = 0.392 |
| SexM |  | *0.022* | *0.037* |  | *-0.228^***^* | *-0.170^***^* |  | *-0.319^*^* | *-0.379^***^* |  | *0.103* | *0.093* |
|  |  | p = 0.995 | p = 0.991 |  | p < 0.0001 | p < 0.0001 |  | p = 0.002 | p < 0.0001 |  | p = 0.794 | p = 0.819 |
| NGMV |  |  | *0.123^*^* |  |  | *0.485^***^* |  |  | *-0.501^***^* |  |  | *-0.080^***^* |
|  |  |  | p = 0.002 |  |  | p < 0.0001 |  |  | p < 0.0001 |  |  | p < 0.0001 |
| R^2^ | 0.023 | 0.023 | 0.030 | 0.005 | 0.051 | 0.150 | 0.039 | 0.128 | 0.234 | 0.016 | 0.025 | 0.028 |
| F | 0.586 | 0.287 | 0.235 | 0.133 | 0.644 | 1.355 | 1.011 | 1.764 | 2.342 | 0.398 | 0.307 | 0.218 |
|  | SDMT | | | 9HPTr | | | SMSW speed | | | EDSS | | |
| Age | *0.151* | *0.144* | *0.200* | *-0.073^***^* | *0.007^***^* | *0.101^***^* | *-0.197^***^* | *-0.086^***^* | *-0.163^***^* | *0.125^***^* | *0.089^**^* | *0.006* |
|  | p = 0.435 | p = 0.497 | p = 0.371 | p < 0.0001 | p < 0.0001 | p < 0.0001 | p < 0.0001 | p < 0.0001 | p < 0.0001 | p < 0.0001 | p = 0.001 | p = 0.834 |
| SexM |  | *0.022* | *-0.007* |  | *-0.228^***^* | *-0.276^***^* |  | *-0.319^*^* | *-0.279^*^* |  | *0.103* | *0.146* |
|  |  | p = 0.995 | p = 0.999 |  | p < 0.0001 | p < 0.0001 |  | p = 0.002 | p = 0.005 |  | p = 0.794 | p = 0.711 |
| NThalV |  |  | *0.173* |  |  | *0.287^***^* |  |  | *-0.236^***^* |  |  | *-0.256* |
|  |  |  | p = 0.895 |  |  | p < 0.0001 |  |  | p < 0.0001 |  |  | p = 0.109 |
| R^2^ | 0.023 | 0.023 | 0.050 | 0.005 | 0.051 | 0.125 | 0.039 | 0.128 | 0.179 | 0.016 | 0.025 | 0.084 |
| F | 0.586 | 0.287 | 0.407 | 0.133 | 0.644 | 1.098 | 1.011 | 1.764 | 1.666 | 0.398 | 0.307 | 0.703 |
|  | SDMT | | | 9HPTr | | | SMSW speed | | | EDSS | | |
| Age | *0.151* | *0.144* | *0.152* | *-0.073^***^* | *0.007^***^* | *0.026^***^* | *-0.197^***^* | *-0.086^***^* | *-0.090^***^* | *0.125^***^* | *0.089^**^* | *0.084^*^* |
|  | p = 0.435 | p = 0.497 | p = 0.475 | p < 0.0001 | p < 0.0001 | p < 0.0001 | p < 0.0001 | p < 0.0001 | p < 0.0001 | p < 0.0001 | p = 0.001 | p = 0.002 |
| SexM |  | *0.022* | *-0.086* |  | *-0.228^***^* | *-0.471^***^* |  | *-0.319^*^* | *-0.266* |  | *0.103* | *0.176* |
|  |  | p = 0.995 | p = 0.982 |  | p < 0.0001 | p < 0.0001 |  | p = 0.002 | p = 0.021 |  | p = 0.794 | p = 0.703 |
| NWMV |  |  | *0.204^***^* |  |  | *0.457^***^* |  |  | *-0.100^***^* |  |  | *-0.137^***^* |
|  |  |  | p < 0.0001 |  |  | p < 0.0001 |  |  | p < 0.0001 |  |  | p < 0.0001 |
| R^2^ | 0.023 | 0.023 | 0.054 | 0.005 | 0.051 | 0.203 | 0.039 | 0.128 | 0.136 | 0.016 | 0.025 | 0.039 |
| F | 0.586 | 0.287 | 0.435 | 0.133 | 0.644 | 1.958 | 1.011 | 1.764 | 1.202 | 0.398 | 0.307 | 0.309 |
|  | SDMT | | | 9HPTr | | | SMSW speed | | | EDSS | | |
| Age | *0.151* | *0.144* | *0.222* | *-0.073^***^* | *0.007^***^* | *0.037^***^* | *-0.197^***^* | *-0.086^***^* | *-0.145^***^* | *0.125^***^* | *0.089^**^* | *0.096^**^* |
|  | p = 0.435 | p = 0.497 | p = 0.294 | p < 0.0001 | p < 0.0001 | p < 0.0001 | p < 0.0001 | p < 0.0001 | p < 0.0001 | p < 0.0001 | p = 0.001 | p < 0.00015 |
| SexM |  | *0.022* | *0.030* |  | *-0.228^***^* | *-0.225^***^* |  | *-0.319^*^* | *-0.325^**^* |  | *0.103* | *0.103* |
|  |  | p = 0.995 | p = 0.993 |  | p < 0.0001 | p < 0.0001 |  | p = 0.002 | p = 0.001 |  | p = 0.794 | p = 0.797 |
| logT2C |  |  | *-0.314* |  |  | *-0.119^***^* |  |  | *0.239* |  |  | *-0.025* |
|  |  |  | p = 0.922 |  |  | p < 0.0001 |  |  | p = 0.017 |  |  | p = 0.952 |
| R^2^ | 0.023 | 0.023 | 0.115 | 0.005 | 0.051 | 0.064 | 0.039 | 0.128 | 0.181 | 0.016 | 0.025 | 0.026 |
| F | 0.586 | 0.287 | 1.000 | 0.133 | 0.644 | 0.526 | 1.011 | 1.764 | 1.698 | 0.398 | 0.307 | 0.201 |
|  | SDMT | | | 9HPTr | | | SMSW speed | | | EDSS | | |
| Age | *0.151* | *0.144* | *0.217* | *-0.073^***^* | *0.007^***^* | *0.035^***^* | *-0.197^***^* | *-0.086^***^* | *-0.126^***^* | *0.125^***^* | *0.089^**^* | *0.071* |
|  | p = 0.435 | p = 0.497 | p = 0.279 | p < 0.0001 | p < 0.0001 | p < 0.0001 | p < 0.0001 | p < 0.0001 | p < 0.0001 | p < 0.0001 | p = 0.001 | p = 0.009 |
| SexM |  | *0.022* | *-0.029* |  | *-0.228^***^* | *-0.248^***^* |  | *-0.319^*^* | *-0.291^*^* |  | *0.103* | *0.116* |
|  |  | p = 0.995 | p = 0.993 |  | p < 0.0001 | p < 0.0001 |  | p = 0.002 | p = 0.003 |  | p = 0.794 | p = 0.773 |
| logT2V |  |  | *-0.404* |  |  | *-0.155^***^* |  |  | *0.220^*^* |  |  | *0.103* |
|  |  |  | p = 0.849 |  |  | p < 0.0001 |  |  | p = 0.002 |  |  | p = 0.718 |
| R^2^ | 0.023 | 0.023 | 0.181 | 0.005 | 0.051 | 0.074 | 0.039 | 0.128 | 0.175 | 0.016 | 0.025 | 0.035 |
| F | 0.586 | 0.287 | 1.698 | 0.133 | 0.644 | 0.614 | 1.011 | 1.764 | 1.628 | 0.398 | 0.307 | 0.279 |
|  | ^*^p^**^p^***^p<1e-04 | | | | | | | | | | | |

**Supplementary Table 2.** Regression models in CIS for functional performance measures as dependent variable and stepwise inclusion of Age, Sex and NBV, NGMV, NThalV, NWMV, logarithmic T2 count and logarithmic T2 volume. Given are the *standardized beta coefficients*, R^2^ and respective F-Statistic, ^*^ is signifying significance after correction for multiple testing.
